# Controlled External Thigh Compression: A Feasible Method to Simulate Venous Hemodynamic Alterations Resembling Deep Vein Thrombosis

**DOI:** 10.1007/s10439-026-04014-y

**Published:** 2026-02-16

**Authors:** Rimvydas Eitminavičius, Rytis Jurkonis, Sami Maja, Roni Ahola, Neringa Balčiūnienė, Antti Vehkaoja, Vaidotas Marozas, Andrius Rapalis

**Affiliations:** 1 Biomedical Engineering Institute, Kaunas University of Technology, K. Barsausko St. 59, 51423 Kaunas, Lithuania; 2 Faculty of Medicine and Health Technology, Tampere University, Korkeakoulunkatu St. 3, 33720 Tampere, Finland; 3 Clinic of Neurosurgery, Lithuanian University of Health Sciences, Mickeviciaus St. 9, 44307 Kaunas, Lithuania

**Keywords:** Deep vein thrombosis, Light reflection rheography, Venous hemodynamics, Venous occlusion plethysmography, Venous stenosis

## Abstract

**Purpose:**

Deep vein thrombosis (DVT) poses significant health risks, including potentially fatal pulmonary embolism. Current clinical practice relies heavily on ultrasonography, requiring a skilled specialist. Alternative methods, such as light reflection rheography (LRR) and venous occlusion plethysmography (VOP), are non-invasive and simple; however, studies report limited consistency and standardization. The development of biosignal-based diagnostic tools is constrained by the inherent risks of DVT, including embolization, and challenges in patient recruitment. The ability to simulate DVT-like conditions would aid in developing and testing alternative screening methods. This study aims to present a simulation method of venous hemodynamic alterations resembling deep vein thrombosis using controlled external thigh compression with ultrasonic visualization.

**Methods:**

Data collection with thirty healthy volunteers was conducted in a laboratory using a commercially available system VasoScreen 5000–4000 to record LRR and VOP signals. Vein stenosis at varying levels was induced through controlled external thigh compression under ultrasonic guidance.

**Results:**

The experimental simulation showed statistically significant but small changes in LRR parameters across different stenosis levels. In comparison, VOP results showed greater differences across stenosis levels, with 70% and 100% performing the best. In these cases, 47% and 70% of the measurements, respectively, were below the normal reference limit, with a notably increased outflow time constant, compared to the baseline measurements, where it remained low despite varying venous capacity.

**Conclusion:**

Presented hemodynamic alterations demonstrated to be a feasible option for simulating DVT-like conditions via controlled external pressure on the thigh.

## Introduction

Deep vein thrombosis (DVT) is a pathological condition characterized by the formation of a blood clot in deep veins. DVT occurs unevenly in the limbs, with approximately 90% of cases occurring in the deep veins of the lower limbs and only approximately 10% in the upper limbs [1]. Regardless of location, thrombi are life-threatening because they can dislodge from the vein walls and travel to the lungs, causing pulmonary embolism (PE). Even if PE does not occur, recurrent thrombosis and post-thrombotic syndrome further contribute significantly to morbidity [2]. The annual incidence rate of DVT ranges from approximately 50–120 cases per 100,000 person-years, but varies depending on the country and region [3]. Although not all cases of DVT progress to PE, all of these pathological conditions impose a significant economic burden, with estimates ranging from 7 to 10 $ billion in direct medical costs in the USA [4], and 1.5–13.2 € billion annually across the European Union [5].

DVT usually arises due to one or more factors from the Virchow’s triad [6, 7]: (1) hypercoagulability (may be triggered by certain medications or genetic factors); (2) vein wall damage (may be triggered by trauma or surgery); and (3) blood flow stasis (may be triggered by long-distance traveling or prolonged bed rest). A person may develop proximal or distal DVT (above or below the knee joint, respectively). In turn, a proximal DVT typically presents with more severe symptoms and carries a higher risk of PE [2, 8]. Furthermore, up to 50% of acute DVT cases occur without specific signs or symptoms [2]. Age also represents an important risk factor, as the incidence of DVT increases significantly after 45 years [9].

Currently, duplex ultrasonography is the standard and widely used diagnostic test. It usually incorporates multiple methodologies, including compression ultrasonography with B-mode ultrasonic imaging and Doppler-based blood flow testing. Generally, two approaches to compression ultrasonography for diagnosing DVT are recommended [8]: either a 2- or 3-point scan at specific locations, or scanning the entire leg at intervals of approximately 2 cm [10, 11]. In compression ultrasonography, a non-collapse of a vein under applied pressure indicates a pathology (e.g., DVT). Nevertheless, it is recommended to use a full duplex ultrasound protocol extending from the thigh to the ankle, including Doppler assessment at specific sites, rather than relying solely on limited 2- or 3-point or complete leg compression ultrasonography, thereby allowing the sensitivity and specificity of ultrasonography for detecting proximal DVT to exceed 90% and, in some cases, approach 100% [10]. However, calf veins are fully visible in only about 60–90% of studies [12], and access to veins located above the inguinal ligament, such as the iliac veins, is also challenging [13]. Thus, underscoring not only the importance of professional expertise but also some limitations of the ultrasonic imaging, which in turn opens the way for use of alternative methods in DVT detection.

Alternative methods, such as light reflection rheography (LRR) and venous occlusion plethysmography (VOP), are based on hemodynamic changes in the venous system and can be used for DVT detection. While the LRR method is established and used for assessing venous reflux and chronic venous insufficiency [14], it is less established and used for DVT detection, as a meta-analysis identified only 9 cohorts including the LRR method for DVT detection [15]. The same meta-analysis reported a sensitivity of 94% for proximal and 92% for distal DVT; however, the specificity was only 71%. Meanwhile, the impedance-based VOP method is more popular, and 42 cohorts were identified [15]. The same meta-analysis reported 88% sensitivity and 90% specificity for proximal DVT using the VOP test; however, the sensitivity for distal DVT was only 28%. Numerical simulations of DVT-induced stenosis have shown that even non-occlusive thrombi (with stenosis greater than 50–60%) can be detected using VOP based on strain gauge plethysmography [16]. Although both strain gauge and impedance plethysmography are established methods for VOP, impedance plethysmography has gained popularity in recent years because of its simpler setup. Generally, the VOP test is considered effective for detecting proximal DVT but is less reliable for distal or small (non-occlusive) DVT. The meta-analysis also noted significant discrepancies among studies due to differing methodologies.

The lack of publicly available LRR and VOP datasets and evidence-based models or simulation methods opens a clear gap not only in LRR and VOP research, but also for DVT in general. In this study, it is hypothesized that controlled external thigh compression with ultrasonic visualization may simulate venous hemodynamic alterations resembling deep vein thrombosis. If proven, this study could reduce the existing research gap and create conditions for the further improvement of existing or development of new models and methods, facilitating pilot testing of ideas and concepts based on hemodynamic changes in the venous system of the lower limbs. The aim of this study is to present a simulation method of venous hemodynamic alterations resembling deep vein thrombosis using controlled external thigh compression with ultrasonic visualization.

**Fig. 1:**
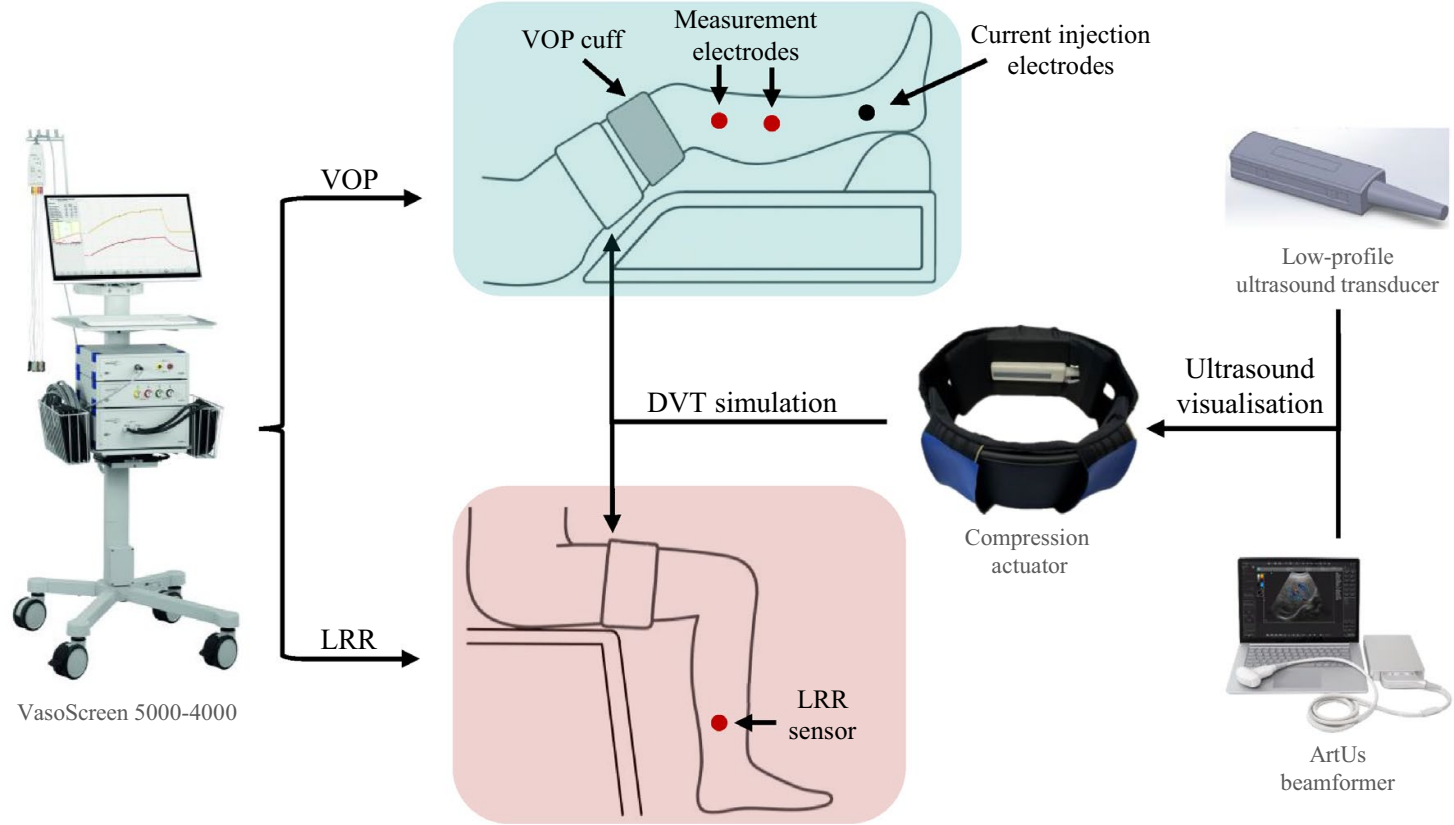
Experimental setup of the study, including used devices and testing positions.

## Materials and Methods

### Study Population and Data Acquisition

Thirty healthy volunteers (15 women) participated in the study. Summarized anthropometric and physiological measurements are provided in Table 1. All participants met the following criteria: (1) age between 18 and 45 years; (2) no chronic pain; (3) no documented or current conditions affecting the cardiovascular and pulmonary systems; (4) no pregnancy or breastfeeding; (5) no professional athletes; and (6) body mass index under 30 kg/m^2^.

**Table 1:** Subject characteristics

Parameter	Mean (SD)
Age (years)	28.5 ($\pm 5.4$)
Weight (kg)	70.3 ($\pm 11.1$)
Height (cm)	175.7 ($\pm 10.7$)
Body mass index (kg/m^2^)	22.6 ($\pm 2.5$)
Thigh circumference (cm)	51.5 ($\pm 3.8$)
Hydration (%)	59.0 ($\pm 7.1$)
Fat mass (%)	19.5 ($\pm 9.7$)
Muscle mass (%)	76.6 ($\pm 9.3$)

Values are presented as mean (standard deviation)

Data collection was conducted indoors at the Biomedical Engineering Institute (Kaunas, Lithuania) in a quiet and temperature-controlled (25.0 °C ± 1.0 °C) laboratory using an experimental setup illustrated in Fig. 1. To perform the LRR and VOP tests, a commercial system VasoScreen 5000-4000 (medis Medizinische Messtechnik GmbH, Germany) was used. Body composition parameters (body weight, muscle mass, fat mass, and hydration) were measured by the segmental body composition analyzer BC-418 (Tanita, Japan).

The LRR sensors with 950-nm wavelength LEDs were placed symmetrically on both legs, approximately 10 cm above the ankle (medial malleolus), on the medial side of each calf (Fig. 1). The LRR device provides two parameters for venous insufficiency; consequently, the LRR signals were preprocessed by applying a Butterworth low-pass filter (4th order, cut-off frequency of 0.25 Hz), and three parameters were calculated: (1) maximum deflection from the baseline *R* [17]; (2) emptying slope $m_{\mathrm{E}}$, calculated during the first 5 s of exercise; and (3) refill slope $m_{\mathrm{R}}$, calculated over 15 s after the end of exercise. The time windows for the emptying and refill slopes differ from those used in previous studies [18]; however, they are not standardized and were selected based on empirical testing to improve measurement consistency within the collected dataset.

During the impedance-based VOP test, the measurement electrodes were placed on both legs on the thickest part of the calf, and the current injection electrodes—just above the ankles, as illustrated in Fig. 1. Impedance plethysmography measurements were performed using a sine wave signal with a frequency of 86 kHz and a current of 1.5 mA. VOP cuffs were placed on the thighs of both legs (just above the knee), and venous blood outflow was stopped by inflating the cuff in three successive steps (maximum pressure 80 mmHg). The device provided six parameters [19]: (1) the arterial inflow *AI* (%/min) is calculated by the slope ($\Delta V/ \Delta t$) of the steepest ascent of each peak of the impedance signal following venous occlusion; (2) the venous capacity *VC* (%) is defined by the difference between the maximal blood volume during venous occlusion and the baseline volume; (3) the venous outflow at 2 s *VO*2*s* (%/min) is calculated by the slope ($\Delta V/ \Delta t$) of the steepest descent during the first 2 s following the VOP cuff deflation; (4) the outflow volume at 3 s *OV*3*s* (%) is defined as the percentage change in limb blood volume that occurs within 3 s after cuff release; (5) the outflow volume at 5 s *OV*5*s* (%) is defined as the percentage change in limb blood volume that occurs within 5 s after cuff release; and (6) the outflow time constant *OTC* (s) is defined as the time constant of the exponential decline in blood volume after VOP cuff release.

Another important component of the experimental setup used for venous compression is the compression actuator with ultrasonic imaging. The integrated air bladder provides tissue compression using piezoelectric air pumps, capable of generating pressures up to 150 mmHg. Ultrasonic visualization is achieved using a portable ArtUS ultrasound beamformer with a low-profile ultrasound transducer (Telemed UAB, Lithuania), integrated into the rigid actuator support mounted on the thigh (ComfTech s.r.l., Italy).

**Fig. 2:**
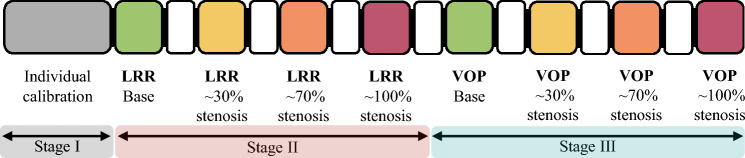
The protocol of the study. Individual calibration, involving manual image labeling, required approximately 10 min. Each LRR test lasted about 1 min, with 3-minute rest intervals between trials. The VOP test lasted approximately 4 min, followed by rest periods of 4 min.

### The Protocol of the Study

The study protocol consisted of nine phases, as depicted in Fig. 2. At the first stage, the compression actuator was mounted on the mid-thigh of the left leg, and the Adductor Canal was identified to confirm that both the femoral artery and vein were clearly visible. Thigh tissue compression with ultrasonic imaging for individual calibration was then performed in a sitting position, as this posture allows the veins to remain maximally filled under normal physiological conditions. After estimating the required pressure levels, the LRR and VOP tests (Stages II and III, respectively) were performed at various stenosis levels induced by external pressure on one leg. Simultaneously, the same measurements were taken on the contralateral (right) leg, which served as a control without DVT simulation.

The LRR measurements were performed in sitting posture and are based on the “muscle pump” mechanism, which is activated by performing specific foot maneuvers (dorsiflexion). Ten consecutive dorsiflexions were performed over 25 s, guided by two-tone sounds produced by the device [20]. A practice test was conducted prior to data collection to allow participants to become acquainted with the exercise and testing setup. The VOP measurements were performed in the supine posture with the legs elevated at a constant height (35 cm, with the heels placed on a soft support). Both LRR and VOP tests were performed according to the manufacturer’s instructions and consistently monitored by the investigators to minimize the risk of hydrostatic differences resulting from postural variations. The rest periods between the LRR and VOP tests were set at 3 and 4 min, respectively, which were sufficient for the signals to return to baseline.

The study was carried out following the ethical principles of the Declaration of Helsinki and with the bioethical approval of the Kaunas Region Biomedical Research Ethics Committee (No. BE-2-67), including informed consent and voluntary participation. Identifiable information was removed from the collected data to ensure the anonymity of the participants.

**Fig. 3:**
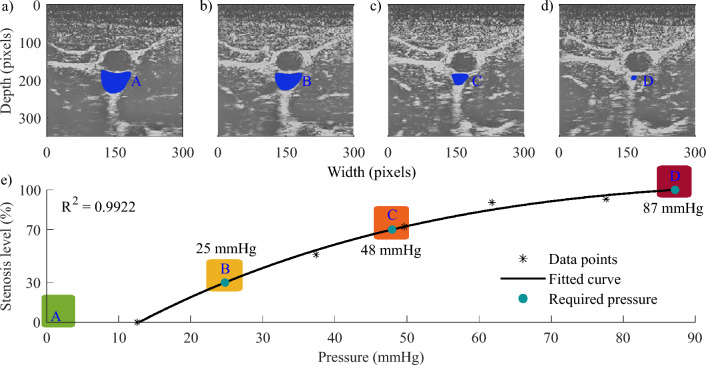
Different stenosis levels of femoral vein in a B-mode view: **a** baseline, **b** 30% stenosis, **c** 70% stenosis, **d** 100% stenosis, and **e** tissue response curve, used to derive the estimated required pressure, after fitting a double-term exponent to vein lumen decrease data points derived from 6 manually labeled B-mode images.

### Experimental Simulation of Venous Hemodynamic Alterations

Individual calibration was performed in a sitting position and was applied across both LRR and VOP tests and all measurements. A calibration in the VOP (supine) position cannot be reliably performed because in that posture, venous pressure is often too low for the veins to be visible, or they collapse under minimal external pressure, making meaningful calibration unfeasible. To achieve a measurable change, occlusion was applied in the sitting position, where the veins are naturally more distended under normal conditions. We hypothesize that stenosis introduced at this level of venous distention best approximates real-world venous closure, such as that caused by an internally forming thrombus, since veins have a finite capacity to expand.

The varying stenosis level of the femoral vein was induced by external pressure on the thigh, applied using the compression actuator. This actuator allows for direct ultrasonic visualization during pressure application, resulting in a controlled and measurable stenosis level. The thigh tissue compression response curve was estimated as follows:

1. Thigh tissues were compressed until full stenosis of the femoral vein was achieved (the maximum required pressure was 130 mmHg) and confirmed in real time via ultrasonic imaging;
2. Six points during the compression process were selected at 15%, 30%, 45%, 60%, 75%, and 95% of the maximum applied pressure;
3. The corresponding six images were labeled using Matlab Image Labeler (Mathworks Inc., USA), with the entire vein area assigned as a mask. The vein cross-section is not necessarily circular, and more often it is elliptically shaped due to uneven pressure distribution (unlike the arteries, which generally maintain a more circular shape) [12]. Therefore, the cross-sectional area is evaluated to assess the level of stenosis.
4. The applied pressure and resulting vein area reduction were fitted using a two-term exponential model, as shown in Fig. 3;
5. From the fitted curve, the required pressures corresponding to $\sim$30%, $\sim$70%, and $\sim$100% stenosis were estimated.

Three pressure levels were selected to simulate varying degrees of stenosis: (1) a 30% stenosis was chosen to represent a non-occlusive or minor thrombus, for exploratory purposes to investigate whether a small narrowing produces measurable effects using these methods; (2) a 70% stenosis was chosen to represent a “detectable” partial occlusion (greater than 50–60% can be detected using the VOP test [16]), incorporating a 10% safety margin to avoid borderline results; and (3) a 100% stenosis level was chosen to represent a full occlusion imitating a severe DVT.

### Statistical Data Analysis

For both LRR and VOP tests, outcomes obtained under varying levels (30%, 70%, and 100%) of femoral vein stenosis were compared with outcomes obtained under standard conditions, without occlusion. The Shapiro–Wilk test was used to assess data normality, and because not all data were normally distributed, the results are summarized using boxplots with medians and quartiles. Statistical analysis was performed using the Wilcoxon signed-rank test for dependent samples, and significance thresholds were set at $p<0.05$, $p<0.01$, and $p<0.001$. To assess the effect size, Cohen’s d for dependent samples was calculated as the mean difference divided by the standard deviation of the differences.

## Results

### General Results of Measured Biosignals and Associated Dependencies

The estimated pressures for different stenosis levels for all subjects were as follows (presented as median and range): 23 mmHg (17–43 mmHg) at 30% stenosis, 44.5 mmHg (26–84 mmHg) at 70% stenosis, and 82 mmHg (43–130 mmHg) at 100% stenosis. Fat mass, muscle mass, and leg length-to-height ratio had a weak correlation with required pressures ranging from − 0.06 to 0.28. While the body mass index had a moderate correlation, ranging from 0.42 to 0.57 across different stenosis levels.

**Fig. 4:**
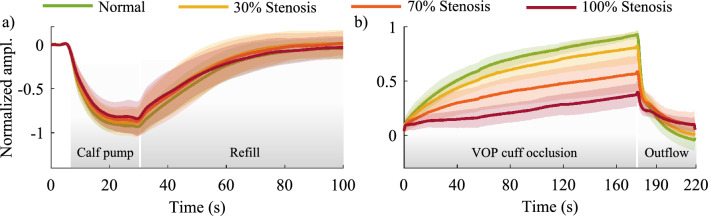
General tendencies of individually normalized **a** LRR and **b** VOP signals (presented as mean and standard deviation).

Figure 4 illustrates the general tendencies of the LRR and VOP signals during different stenosis levels. Each subject’s measurements were normalized to the maximum of the first measurement, and averaged across subjects to visualize changes caused by external pressure. The LRR signals show only minor visual differences between the stenosis levels; nevertheless, a reduced maximum deflection, the slope of the emptying phase, and the slope of the refill phase can be observed. The VOP signals show a clear separation between levels of stenosis, where the relative change of the signal amplitude and outflow slope is reduced with increasing stenosis levels.

**Fig. 5:**
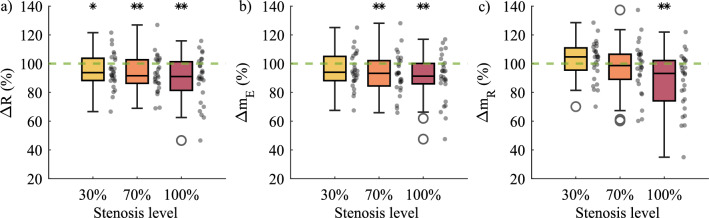
Relative changes in LRR parameters for **a** $\Delta R$, **b** $\Delta m_{\mathrm{E}}$, and **c** $\Delta m_{\mathrm{R}}$ across increasing stenosis levels. All values are expressed relative to the first (non-occluded) measurement, which serves as the baseline and is indicated by a green dashed line at 100%. Significant deviations from this baseline are marked with “*” ($p<0.05$) and “**” ($p<0.01$), based on statistical analysis of raw (non-normalized) data.

**Fig. 6:**
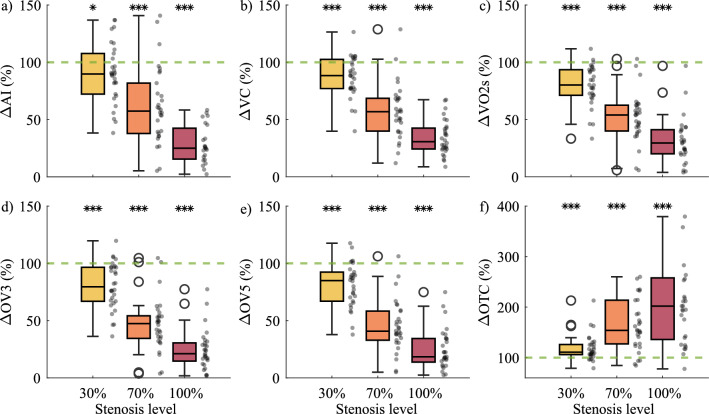
Relative changes in VOP parameters for **a** $\Delta AI$, **b** $\Delta VC$, **c** $\Delta VO2s$, **d** $\Delta OV3$, **e** $\Delta OV5$, and **f** $\Delta OTC$ across increasing stenosis levels. All values are expressed relative to the first (non-occluded) measurement, which serves as the baseline and is indicated by a green dashed line at 100%. Significant deviations from this baseline are marked with “*” ($p<0.05$) and “***” ($p<0.001$), based on statistical analysis of raw (non-normalized) data.

### LRR Parameter Analysis

As shown in Fig. 5, during the LRR tests a 30% stenosis decreased the median of $\Delta R$ (− 6.3%) and $\Delta m_{\mathrm{E}}$ (− 6.0%); however, the $\Delta m_{\mathrm{R}}$ increased by 4.7%. A 70% stenosis decreased the median of $\Delta R$ (− 8.4%), $\Delta m_{\mathrm{E}}$ (− 6.8%), and $\Delta m_{\mathrm{R}}$ (− 1.4%). A 100% stenosis decreased the median of $\Delta R$ (− 9.0%), $\Delta m_{\mathrm{E}}$ (− 8.7%), and $\Delta m_{\mathrm{R}}$ (− 6.8%). Three subjects were excluded from the analysis due to poor signal quality. Although all three parameters show a statistically significant difference at the 100% stenosis level, and $\Delta R$ and $\Delta m_{\mathrm{R}}$ parameters also at 70%, the results are not consistent with increasing stenosis level, making the distinction between normal and abnormal cases difficult.

### VOP Parameter Analysis

As shown in Fig. 6, during the VOP tests, a 30% stenosis decreased the median of $\Delta AI$ (− 10.2%), $\Delta VC$ (− 11.6%), $\Delta VO2s$ (− 19.9%), $\Delta OV3$ (− 20.4%), and $\Delta OV5$ (− 15.1%), while the median of $\Delta OTC$ increased by 10.9%. A 70% stenosis decreased the median of $\Delta AI$ (− 42.6%), $\Delta VC$ (− 43.2%), $\Delta VO2s$ (− 46.0%), $\Delta OV3$ (− 52.7%), and $\Delta OV5$ (− 59.3%), while $\Delta OTC$ increased by 53.9%. A 100% stenosis decreased the median of $\Delta AI$ (− 75.0%), $\Delta VC$ (− 69.3%), $\Delta VO2s$ (− 70.4%), $\Delta OV3$ (− 78.9%), and $\Delta OV5$ (− 81.6%), while $\Delta OTC$ increased by 102.1%. All parameters showed statistically significant differences across all stenosis levels compared to the first measurement without occlusion.

### Validation of Simulation Methodology

**Fig. 7:**
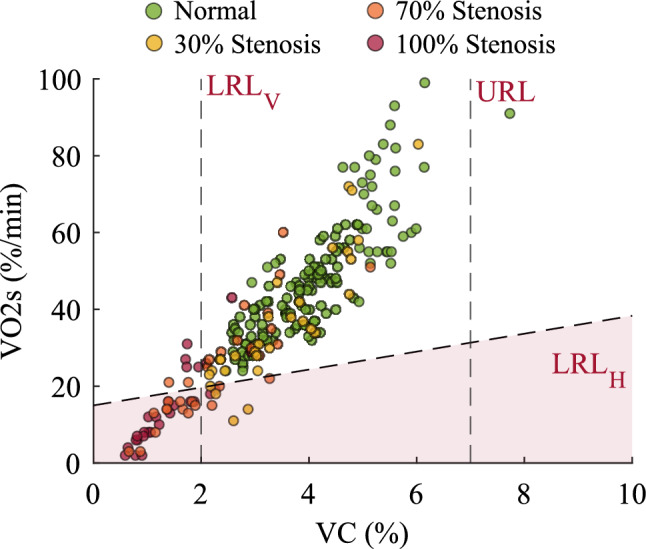
Scatter plot of *VC* and *VO*2*s* parameters with thresholds for abnormal values. Abnormal values for *VC* are less than $LRL_{\mathrm{V}}$ and greater than *URL*, for *VO*2*s*—less than $LRL_{\mathrm{H}}$.

The DVT simulation methodology was validated using several VOP test metrics. *VO*2*s* parameter is plotted against *VC* in Fig. 7, with a few thresholds for abnormal values (potentially indicative of DVT) selected based on the literature. For *VC*, abnormal values are defined as less than 2% (vertical lower reference level $LRL_{\mathrm{V}}$) or greater than 7% (uper reference level $URL$), while for *VO*2*s*, values lower than $7/3 \times VC + 15$ %/min (horizontal lower reference level $LRL_{\mathrm{H}}$) [19]. To provide more results and obtain a broader view of the analysis, measurements from the right leg were also included in the analysis. None of the measurements without occlusion fell below ($LR L_{\mathrm{H}}$) limit. However, one subject’s right leg measurements were excluded due to poor signal quality. For 30% stenosis, four cases (13.3%) fell below $LRL_{\mathrm{H}}$ limit, indicating abnormal values (unexpected result), as the literature suggests that only stenoses greater than 50–60% should be detectable using the VOP. At 70% and 100% stenosis levels, 14 and 21 cases, respectively, fell below $LRL_{\mathrm{H}}$ limit, representing 46.7% and 70.0% of the measurement groups at those levels. Additionally, 2 and 4 cases, were on the left side of the vertical lower reference limit $LRL_{\mathrm{V}}$ at 70% and 100% stenosis, respectively.

**Fig. 8:**
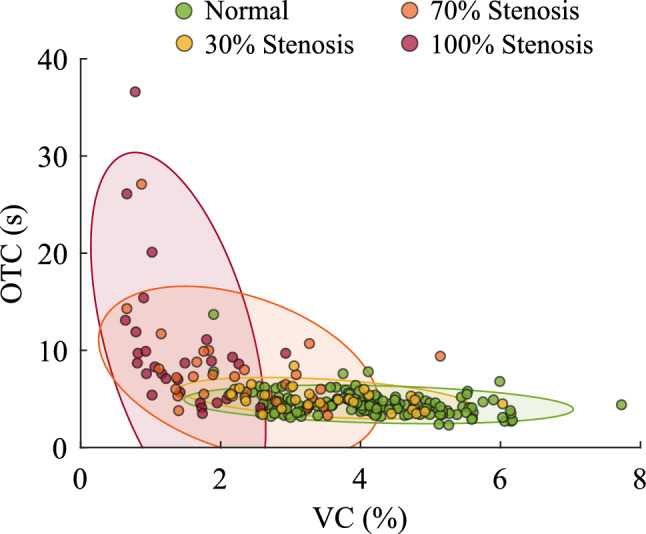
Scatter plot of *VC* and *OTC* parameters. The ellipses represent the 95% confidence bounds of measurements without occlusion and with different stenosis levels.

Figure 8 shows *OTC* plotted against *VC*, with 95% confidence ellipses for normal (no stenosis) and abnormal (different stenosis levels) cases. In normal cases, *OTC* remains low despite increasing *VC*; conversely, abnormal cases exhibit elevated *OTC* values even at low *VC*. The majority of normal measurements cluster around 4 s, whereas abnormal (70% and 100% stenosis) measurements are centered around 8 s and 11 s, respectively.

## Discussion

The present study investigated the feasibility of altering venous hemodynamics in the lower limb to simulate DVT-like conditions. The simulation was performed directly on human subjects, as the technical feasibility of in vitro simulation is currently limited, because of the high complexity of the lower limb venous system. A compression actuator, combined with real-time ultrasonic visualization, serves as a novel hemodynamic testbed and enables detailed assessment of the LRR and VOP methods. To the best of the authors’ knowledge, there are currently no non-invasive, fully evidence-based models for simulating DVT-like conditions. This study integrates ultrasonography with LRR and VOP methods, providing new insights into these techniques and enabling phenomenological simulation of altered venous hemodynamics through direct experimental observations.

Although the analyzed parameters for LRR show statistically significant changes, primarily at the 70% and 100% stenosis levels, the effect sizes were consistently small to moderate (Cohen’s d varied from − 0.06 to 0.52), indicating limited but potentially meaningful physiological changes. The LRR signals obtained from this experimental simulation do not appear as suppressed as those reported in studies involving occlusive venography-confirmed DVT cases [17]. However, as noted by Mitrani et al., the maximum deflection parameter exhibits a statistically significant difference [21], which aligns with the findings of this experimental study. Still, the question remains whether the DVT simulation, which induces stenosis via external pressure, accurately reflects actual thrombosis. The simulation may not fully represent the complexity of actual thrombotic occlusion due to the relatively high-pressure environment created by the hydrostatic column of venous blood in the sitting posture and the active calf muscle pump [22], combined with the naturally compliant veins of healthy subjects. Another possibility is that the variability in how each subject performed the exercise exceeded the true effect size. Notably, in some cases, the effect size was substantial, with one subject showing a reduction of approximately 60% across all three parameters. Furthermore, intra-subject analysis of the right leg (which underwent no occlusion) revealed a relatively high coefficient of variation. The mean CV across the study sample was 9.3% for $R$, 9.8% for $m_{\mathrm{E}}$, and 11.7% for $m_{\mathrm{R}}$, suggesting considerable variability in exercise performance between measurements. This study did not employ any methods to monitor pump-exercise consistency (e.g., IMU sensor), and this could be an area for future research.

A recent study exploring LRR parameters [18] describes the simulation of mild and severe DVT by inflating a thigh cuff to 100 and 150 mmHg, respectively. However, there is no evidence provided regarding the resulting stenosis level or how the deep veins are affected. In contrast, the present study allows for direct ultrasonic visualization during femoral vein compression, with the median maximum pressure required to completely occlude the vein measured at 82 mmHg (43–130 mmHg). However, the pressure values are most likely not directly comparable, as the compression in the present study is performed only from the posterior side. It has to be noted that a subject-specific calibration is necessary to determine optimal pressure levels that account for anatomical variability among individuals.

The results of this phenomenological DVT simulation study are partially consistent with findings from a numerical modeling study, which suggested that stenoses greater than 50–60% should be detectable using strain gauge plethysmography [16]. The parameter analysis in the current study indicates that even a 30% stenosis produces statistically significant differences compared to conditions without stenosis, as measured by impedance-based plethysmography. However, distinguishing between normal and abnormal cases at this level remains challenging, as the altered parameter values may still fall within the normal range. This highlights the potential value of tracking relative changes in parameters over time, or employing bilateral leg comparisons, since bilateral DVT is rare [23], rather than relying solely on absolute thresholds. Such approaches may offer earlier insight into subtle hemodynamic alterations. The VOP signals obtained from this experimental simulation are consistent with the literature [24], which reports that normal outflow after VOP cuff release is very rapid, whereas abnormal outflow is significantly slower. Additionally, the obtained results align with the findings of the study, where a VOP test was performed on patients with venography-confirmed DVT [25]. Showing that for patients with acute proximal DVT, venous capacity is greatly reduced, whereas the outflow time constant tends to increase. Also, abnormal cases were scattered rather than tightly grouped as the normal ones, illustrating the inherent variability between individuals and explaining discrepancies in detection across stenosis levels of our results. All VOP parameters showed predominantly large to very large effects (Cohen’s d varied from − 0.81 to 2.59), reflecting substantial stenosis-induced changes.

Lastly, it should be noted that during both the acute and chronic stages of thrombosis, vein walls thicken and become stiffer [26]. In this simulation, however, the vein walls remain unchanged and are naturally compliant. The femoral vein, situated in the adductor canal, is surrounded by soft tissue, which permits expansion during VOP cuff release and dorsiflexion exercises (confirmed by ultrasonography). This is presumably not the case in real thrombosis, where the thrombus forms intraluminally and is accompanied by venous wall stiffening, limiting the vein’s capacity to expand, consequently resulting in higher hemodynamic changes than those caused by an externally induced stenosis.

Dai et al. [27] and Rohan et al. [28] described the asymmetric compression, which is similar to our anterior–posterior compression approach. In the present study, the vein response for up to 60% of maximum compression pressure follows the tendency in a similar way described by the two studies, where the vein cross-section tends to elongate with increasing pressure (in our case, the median eccentricity changed from  0.48 to  0.6); however, after that, the eccentricity is quite scattered and does not follow a clear pattern (drops back to  0.5), which could be a result of unique compression by our setup which is different from the ordinary compression done by just ultrasound transducer.

Correlations between multiple subject characteristics (fat mass, muscle mass, leg length-to-height ratio, and hydration) and the required pressures were examined. The results demonstrated very weak to weak correlations. This is most likely because the majority of healthy volunteers had normal hydration levels, with a median of 60% (range: 46–71%), leaving insufficient variability to assess the impact of hydration. Inclusion of dehydrated or overhydrated subjects would likely provide more meaningful insights. In contrast, body mass index showed a moderate correlation (range: 0.42–0.57) with the required pressure. The body mass index itself was strongly correlated with thigh circumference ($0.78$), suggesting that leg size, rather than the muscle mass or fat mass, influences the required pressure more.

Limitations of this study include the following: (1) Ultrasonic imaging variability: good image quality remains a challenge due to the inherent limitations of ultrasonography. Labeling the cross-sectional area is subject to potential error, as inconsistent image quality can affect annotation accuracy. (2) Compression application: the usage of the compression actuator requires meticulous setup to ensure stable and uniform compression. This process can introduce variability if not carefully controlled. (3) Use of external pressure: compression of superficial veins most likely affects the recorded signals; however, this limitation is inherent to externally induced stenosis and is unlikely to be fully resolved, unless applied locally. Additional limitations, related to anatomy and subjectivity, include (1) differences in individual calibration and VOP test positions: individual calibration is performed in a sitting position, while the VOP test is conducted in a supine position. This can introduce some inaccuracy; however, calibration in the VOP position is not feasible, as the veins are completely or nearly collapsed. (2) Variation in how the pump exercise is performed: differences in participant performance during the LRR exercise may introduce variability in the effectiveness of the exercise across repeated measurements. In order to eliminate or reduce the described limitations, future work should focus on applying this workflow to broader populations, improving ultrasound imaging quality, and exploring both existing and novel signal parameters. These steps will help refine the methodology and expand its utility as a controlled experimental platform for studying venous hemodynamics.

In conclusion, the proposed DVT hemodynamic simulation demonstrated to be a feasible option to simulate DVT-like conditions via controlled external pressure on the thigh. This allows the simulation to be used in pilot testing measurement methodologies based on the venous hemodynamics of the lower limb. Also, it must be noted that the limited sample size and relatively young age of the cohort used in this study may limit generalizability, as venous anatomy and hemodynamics (e.g., venous wall stiffening) change with aging. Therefore, this should be considered in future studies.

## Data Availability

The datasets collected during the study will be available from the corresponding author after the end of the ThrombUS+ project upon reasonable request.
